# Antibiosis and *bmyB* Gene Presence As Prevalent Traits for the Selection of Efficient *Bacillus* Biocontrol Agents against Crown Gall Disease

**DOI:** 10.3389/fpls.2017.01363

**Published:** 2017-08-14

**Authors:** Olfa Frikha-Gargouri, Dorra Ben Abdallah, Ilhem Bhar, Slim Tounsi

**Affiliations:** Biopesticides Laboratory, Centre of Biotechnology of Sfax, Sfax University Sfax, Tunisia

**Keywords:** *Agrobacterium tumefaciens*, crown gall disease, biological control, *Bacillus*, lipopeptides, antibacterial

## Abstract

This study aimed to improve the screening method for the selection of *Bacillus* biocontrol agents against crown gall disease. The relationship between the strain biocontrol ability and their *in vitro* studied traits was investigated to identify the most important factors to be considered for the selection of effective biocontrol agents. In fact, previous selection procedure relying only on *in vitro* antibacterial activity was shown to be not suitable in some cases. A direct plant-protection strategy was performed to screen the 32 *Bacillus* biocontrol agent candidates. Moreover, potential *in vitro* biocontrol traits were investigated including biofilm formation, motility, hemolytic activity, detection of lipopeptide biosynthetic genes (*sfp, ituC* and *bmyB*) and production of antibacterial compounds. The obtained results indicated high correlations of the efficiency of the biocontrol with the reduction of gall weight (*p* = 0.000) and the antibacterial activity *in vitro* (*p* = 0.000). Moreover, there was strong correlations of the efficiency of the biocontrol (*p* = 0.004) and the reduction in gall weight (*p* = 0.000) with the presence of the *bmyB* gene. This gene directs the synthesis of the lipopeptide bacillomycin belonging to the iturinic family of lipopeptides. These results were also confirmed by the two-way hierarchical cluster analysis and the correspondence analysis showing the relatedness of these four variables. According to the obtained results a new screening procedure of *Bacillus* biocontrol agents against crown gall disease could be advanced consisting on two step selection procedure. The first consists on selecting strains with high antibacterial activity *in vitro* or those harbouring the *bmyB* gene. Further selection has to be performed on tomato plants *in vivo*. Moreover, based on the results of the biocontrol assay, five potent strains exhibiting high biocontrol abilities were selected. They were identified as *Bacillus subtilis* or *Bacillus amyloliquefaciens*. These strains were found to produce either surfactin or surfactin and iturin lipopeptides. In conclusion, our study presented a new and effective method to evaluate the biocontrol ability of antagonistic *Bacillus* strains against crown gall disease that could increase the efficiency of screening method of biocontrol agents. Besides, the selected strains could be used as novel biocontrol agents against pathogenic *Agrobacterium tumefaciens* strains.

## Introduction

*Agrobacterium tumefaciens* is the causal agent of the crown gall, one of the most economically important diseases of crops (Kennedy and Alcorn, 1980). This strain produces tumours on a large variety of plants including those economically important (Otten et al., 2008). The disease is rarely fatal, unless when affecting young or stressed plants. It is widespread in nurseries of fruit trees and ornamental plants. The infections lead to the loss of plant vigour and/or the reduction in crop yield. In nurseries, affected plants must be culled and discarded. This disease is difficult to control by the use of chemical pesticides and can spread to other plants easily. As alternative to these pesticides, biological control represents an environmentally sound and an attractive method for plant protection. Two commercially biocontrol agents were developed to control crown gall disease. These were *Agrobacterium rhizogenes* strain K84 and its derivative K1026 producing the agrocin 84, an antibiotic like product, with specific antibacterial activity toward *Agrobacterium* strains. However, these biocontrol agents failed to control tumour development on grapes and some other economically important plants (Otten et al., 2008). Thus, there is a need to search alternative antagonistic bacterial strains able of controlling *A. tumefaciens*. Various bacteria were tested as biological control agents against the crown gall disease. Of these, *Bacillus* strains were reported to be efficient in reducing gall formation on grapevine and on tomato plants (Eastwell et al., 2006; Gupta and Khosla, 2007; Rhouma et al., 2008; Hammami et al., 2009; Ben Abdallah et al., 2015; Frikha-Gargouri et al., 2017).

*Bacillus*-based biological control agents account for about half of commercially available bacterial biological control agents. They belong to one of the most studied genus for the control of plant diseases. They have often been reported to be among the most beneficial microorganisms used as antagonists against phytopathogenic bacteria, fungi and insects. In addition to being ubiquitous, *Bacillus* species are considered safe organisms. They produce resistant spores and several broad-spectrum antibiotic compounds. All these characteristics confer to this genus exceptional ecological advantages and allow their long-term storage and relatively easy commercialization. Their protective effect could rely on different mechanisms making them suitable for agricultural applications as biocontrol agents of plant diseases including antagonism, competition, induction of plant defence responses and growth promotion (Haas and Défago, 2005). Regarding antibiosis, *Bacillus* genus produces various bioactive compounds (lipopeptides, polyketides and bacteriocins) that have been suggested to play important roles in plant disease control. Among these compounds, lipopeptides encompassing the surfactin, the iturin and the fengycin families showed potent activities against a wide variety of microorganisms (Peypoux et al., 1999; Bonmatin et al., 2003; Ongena and Jacques, 2008). The gene clusters, encoding peptide synthetases for the surfactin (*srf*), iturin (*itu*), bacillomycin (*bmy*) and fengycin (*fen*) have been studied (Tosato et al., 1997; Tsuge et al., 2001; Koumoutsi et al., 2004). Moreover, the antibacterial activities of lipopeptides have been studied. Only the surfactin and the iturin families display antibacterial activity (Ongena and Jacques, 2008). Surfactin and iturin production *in vivo* have been implicated in the reduction of infection by *Pseudomonas syringae* in *Arabidopsis* plants (Bais et al., 2004) and those caused by *Xanthomonas campestris* pv. *cucurbitae* and *Pectobacterium carotovorum* subsp. *carotovorum* in detached melon leaves, respectively (Zeriouh et al., 2011). These lipopeptides are known to act synergistically (Maget-Dana et al., 1992; Tanaka et al., 2015). Besides the antimicrobial activity, lipopeptides have been involved, in the attachment to plant surfaces, to the formation of biofilms and to the induction of resistance against phytopathogens (Hofemeister et al., 2004; Ongena et al., 2007; Chen et al., 2013).

In our previous studies, promising bio-control against the crown gall disease, strains 32a and 39b, were selected based on their high biosurfactant and antibacterial activities *in vitro* (Ben Abdallah et al., 2015; Frikha-Gargouri et al., 2017, unpublished data). Despite their high activities, the biocontrol results indicated the effectiveness of strain 32a, however, that of strain 39b was only partial. Such failure in the biocontrol against other diseases was also reported for some strains isolated using *in vitro* tests such as antibiosis (Fravel, 1988; Inam-ul-Haq et al., 2003; Rajkumar et al., 2005; Ran et al., 2005). Thus, correlating *in vitro* and *in vivo* experiments should be interpreted with caution. *In vitro* screening is often preferred as selection mechanism since most commercial biopesticides exhibits antibiosis as the main mechanism of action. Moreover, this method is easy, fast, inexpensive to perform, and permits massive screening of several strains. On the contrary, the screening using *in vivo* experiments through a plant protection strategy is expensive, difficult to perform, time-consuming, and only allowed the study of a limited number of bacterial strains. Thus in our study, we aimed to develop a new and effective method to evaluate antagonistic *Bacillus* strains against crown gall disease that could increase the efficiency of screening method of biocontrol agents. A plant protection strategy as well as *in vitro* investigations were performed for all the strains to identify the most important factors to be considered for the selection of biocontrol agents against crown gall disease.

## Materials and Methods

### Isolation and Characterization of *Bacillus* Strains

#### Isolation of *Bacillus* Strains

Spore forming bacteria from different soil and rhizospheric samples were isolated after treatment at 80°C for 10 min. The resulting spores were then plated on LB agar medium and were allowed to grow for 24 h. Random colonies were then picked from the agar plates. These colonies were purified by repeated streaking of single colonies on fresh agar plates and then stored at -80°C.

#### Culture Media

Antagonistic *Bacillus* strains were cultured in the OM medium (Mezghanni et al., 2012) for the production of antibacterial compounds.

#### Screening of the Antagonistic *Bacillus* Strains

Assessment of the antagonistic *Bacillus* strains against *A. tumefaciens* strain C58 was performed by inoculating the pathogenic strain on the surface of agar plates. Then, the *Bacillus* sp. strains were inoculated on the surface of LB agar plates using sterile toothpicks. After incubation for 24 h at 30°C, the antibacterial compound production was indicated by a clear zone of inhibition around the bacterial growth. Strains producing antibacterial compounds were selected for further experiments.

#### Biofilm Formation

Biofilm formation was assessed using the microtiter plate assay (O’Toole et al., 2000). Selected bacterial strains were grown in LB medium at 30°C. *Bacillus* cells were then diluted to an optical density at 595 of 0.01 in LB medium. Hundred microlitre of the diluted cells were allocated to each well of the 96-well polystyrene microtiter plates. The inoculated plates were incubated at stationary condition at 30°C for 48 h. Staining of adhered cells was performed by the addition of 100 μl cristal violet solution (0.1%) at room temperature for 20 min. Excess of the cristal violet solution was then removed, and the wells were rinsed twice with water. After that, 100 μl of dimethyl sulfoxide (DMSO) was added to each dry well. The samples were set for 20 min, and their optical densities at 620 nm were measured on a plate reader. *Escherichia coli* Top10 was included as the negative control. This experiment was repeated at least twice.

#### Motility Assays

Swimming and swarming abilities were tested in king B medium diluted 1/20 in Milli-Q water containing 0.3 and 0.5% agar, respectively. For all motility assays, the strains were stab-inoculated on the surface of agar plates. Motility was recorded after 48 h of incubation. Assays were repeated at least twice.

#### Hemolytic Activity Assays

The hemolytic activity assay was carried out using the Columbia blood agar medium. The strains were stab-inoculated on the surface of agar plates. The clearing zones were recorded after 48 h of incubation. Assays were repeated at least twice.

#### DNA Isolation and Detection of Lipopeptide Biosynthetic Genes

Genomic DNA was isolated from the *Bacillus* strains by standard protocols (Sambrook and Russell, 2001). Biosynthetic genes involved in the production of surfactin, iturin, and bacillomycin were amplified by PCR from genomic DNA. Primer pairs SFP-F1 (ATGAAGATTTACGGAATTTA) SFP-R1 (TTATAAAAGCTCTTCGTACG); ITUC-F1 (CCCCCTCGGTCAAGTGAATA) ITUC-R1 (TTGGTTAAGCCCTGATGCTC) and BMYB-F (GAATCCCGTTGTTCTCCAAA) BMYB-R (GCGGGTATTGAATGCTTGTT) were used, respectively (Chung et al., 2008). The PCR mixture composed of 1× PCR buffer; 0.2 mM each deoxynucleoside triphosphate (dATP, dGTP, dCTP, and dTTP); 0.5 mM of each primer; 1.5 U of DNA polymerase; and 50 ng of template DNA. The Gene-Amp PCR System 9700 (Perkin Elmer Cetus) was used for PCR amplification. The PCR includes an initial cycle at 94°C for 5 min; 35 cycles of denaturation at 94°C for 30 s, annealing at 50, 50, 55°C, respectively, for surfactin, iturin and bacillomycin for 1 min, and elongation at 72°C for 1 min; with a final extension at 72°C for 7 min. The amplifications were analysed by electrophoresis of 5 μl in an 1.5% agarose gel followed by ultraviolet visualisation.

### Characterization of the Antibacterial Compounds

#### Production of the Antibacterial Compounds and Detection of the Antibacterial Activity

Cultures of *Bacillus* strains were performed at 30°C with shaking at 200 rpm. An initial optical density of 0.1 was used to inoculate 50 ml of the OM medium in 250 ml Erlenmeyer flasks. The antibacterial activity in the culture supernatant was tested after 48 h of culture by the well diffusion assay. For that, agar plates were inoculated with the *Agrobacterium* strain suspension. Then, wells were created and used to apply 50 μl of culture supernatant. Clear zone of inhibition around the bacterial growth observed after incubation for 24 h at 30°C indicating the antibacterial compound production was assessed. The extent of inhibition zone was measured. This experiment was repeated at least three times.

#### Effect of Proteinase K on the Antibacterial Activity

Supernatants with antibacterial activity against *A. tumefaciens* C58 were incubated at 37°C for 1 h with the proteinase K enzyme (1 mg/ml). This latter was then inactivated by boiling for 10 min. Supernatant without the addition of the proteinase K served as negative control. They were subjected to the same treatments as the positive control. All samples were then tested for their antibacterial activity against *A. tumefaciens* C58.

### *In Vivo* Biocontrol Test of the Antagonistic Strains toward *A. tumefaciens*

Efficiency of the antagonistic strains was assessed on tomato plants (*Lycopersicum esculentum*). The test consists in controlling the specific gall formation after 1 month of inoculation of the pathogenic strain *A. tumefaciens* C58 with and without treatments with *Bacillus* strains. For that, wounds were generated on the stems of tomato plants using a sterile scalpel. These wounds were then inoculated with the pathogens (10^8^ CFU) with or without *Bacillus* strains (10^8^ CFU). Untreated plants served as negative control. Positive control consists of plants inoculated only with the phytopathogenic bacteria C58. For each treatment, four plants were tested and each plant includes three wounds. Plants were maintained at 22–26°C, with a natural photoperiod. The stems were checked for galls development periodically. The number of formed galls and their weight were determined after 1 month of infection.

### Characterization of Potent Antagonistic Strains

#### Isolation and Identification of Lipopeptides Using HPLC Analysis

Cell-free culture was adjusted to pH 2 with 6 M HCl in order to precipitate the lipopeptides and was then kept overnight at 4°C. After centrifugation, the precipitate was extracted with methanol and then concentrated. The concentrated extract was then purified on a C_18_ SPE column. The eluted fraction was concentrated again and then analysed by HPLC using as mobile phase Milli-Q water containing 0.1% trifluoroacetic acid and acetonitrile. The elution was performed using a gradient of 40–100% acetonitrile (56 min) at a flow rate of 0.6 mL/min. UV detection used a wavelength of 214 nm. Three main groups of peaks were observed at elution times comparable with those observed for standard lipopeptides (Surfactin, iturin and fengycin).

#### Identification of the Potent Antagonistic Bacteria

Molecular characterization was performed by 16S rRNA sequencing. Primers Fd1 (5′-AGAGTTTGATCCTGGCTCAG-3′) and Rd1 (5′-AAGGAGGTGATCCAGCC-3′) were used for PCR amplification (Weisburg et al., 1991). The PCR mixture consisted of 1× PCR buffer; 0.2 mM each deoxynucleoside triphosphate (dATP, dGTP, dCTP, and dTTP); 0.5 mM of each primer; 1.5 U of DNA polymerase (Promega, France); and 50 ng of template DNA. The following conditions were used for PCR amplification: an initial cycle at 94°C for 3 min, followed by 30 cycles of denaturation at 94°C for 30 s, annealing at 53°C for 1 min and elongation at 72°C for 2 min. The resulting PCR fragments were purified and used for sequencing.

### Statistical Methods

One way analysis of variance (ANOVA) and correlations were analysed using the Statistical Package for the Social Sciences (SPSS V.11; SPSS Inc., Chicago, IL, United States). The mean values among the treatments were compared using the Duncan’s multiple range test at the 5% level of significance (*p* = 0.05).

The R programme was used for the principal component analysis and the two-way hierarchical cluster analysis using the heatmap and the pca methods. The principal component analysis was performed for the studied traits as well as for the *Bacillus* strains to assess the relationships between them. Moreover, the two-way hierarchical cluster analysis based on Ward’s method was performed. It allowed the construction of clusters of the *Bacillus* isolates and the various studied traits to reveal those with similar patterns.

## Results

### Isolation and Characterization of *Bacillus* Strains

A thousand of sporulating *Bacillus* strains was isolated from environmental samples. All isolates were subjected to antibacterial activity testing against *A. tumefaciens* strain C58. Thirty strains were found to form haloes of *Agrobacterium* cell lysis around the antagonist growth zone indicating the production of antibacterial compounds. Two other strains without detectable activity were also included. These strains were retained for further experiments (**Table 1**).

**Table 1:** Characterization of the colony activity, biofilm formation, motility and hemolytic activity of the *Bacillus* strains.

Colony activity	Biofilm formation	Motility	Hemolysis	Strain
+	+	+	+	12b, 13b, 14b, 28a, 28b, 32a, 33b, 35b, 38b, 39b, 44a, 44b, 45a, 46b, 53a, 53b, 54a, 56b, 63a, 63b, 66a, 73b, 76b, 85a, 87a
-	+	+	+	57a
-	-	-	-	59a
+	+	+	-	62b, 72b, 73a, 83a
+	-	-	-	69a

#### Biofilm Formation, Motility Assays and Hemolytic Activity

Characterization of the 32 selected strains of *Bacillus* spp. indicated that all of them were able to form a biofilm and to efficiently colonise the media after 48 h of incubation, except strains 59a and 69a. These two strains, as well as strains 62b, 72b, 73a and 83a, were unable to lyse red blood cells. The results are presented as **Table 1**. Statistical analysis indicated that there were correlations between biofilm formation, motility and hemolysis (*p* < 0.05).

#### Detection of the Lipopeptide Biosynthetic Genes

**FIGURE 1:**
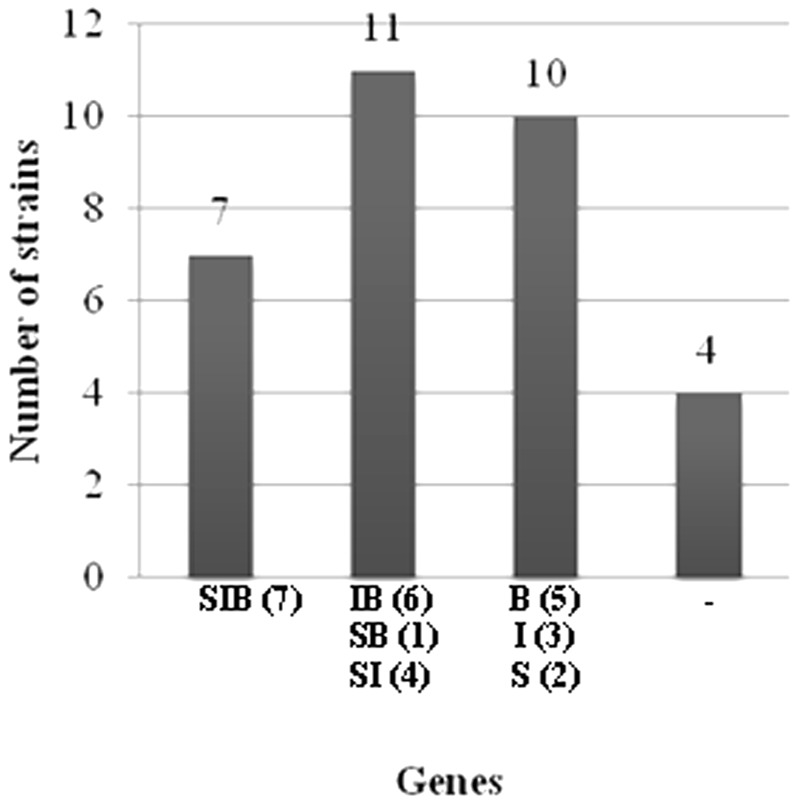
Classification of strains according to the number of the biosynthetic genes detected. S: *sfp* gene, I: *ituC* gene, B: *bmyB* gene, –: no gene detected, parenthesis: number of strains.

All strains were tested for the presence of genes involved in the biosynthesis of surfactin, iturin and bacillomycin. The amplifications of these genes with the used primer pairs generated PCR products with expected sizes of 675, 594 and 370 bp, respectively (Data not shown). Based on the presence or the absence of antibiotic biosynthetic genes, the studied strains were classified (**Table 2**). Seven out of the thirty-two strains harbour all the three genes and four of them had no one. Eleven strains had two antibiotic genes and ten had only one (**Figure 1**). The most frequently detected genes were the *ituC* (20 times) and the *bmyB* (19 times), followed by the *sfp* (14 times). A correlation between the detection of *ituC* gene with biofilm formation and motility was observed (*p* < 0.45).

**Table 2:** Classification of strains according to their biosynthetic genes detected.

*sfp*	*ituC*	*bmyB*	Strain	Number of strains
+	+	+	32a, 35b, 39b, 45a, 57a, 66a, 83a	7
-	+	+	28a, 28b, 33b, 56b, 63a, 73b	6
+	-	+	54a	1
+	+	-	38b, 44a, 44b, 62b	4
-	-	+	12b, 14b, 46b, 72b, 73a	5
-	+	-	53b, 76b, 87a	3
+	-	-	53a, 85a	2
-	-	-	13b, 59a, 63b, 69b	4

### Characterization of the Antibacterial Compounds

#### Production of the Antibacterial Compounds and Detection of the Antibacterial Activity

**FIGURE 2:**
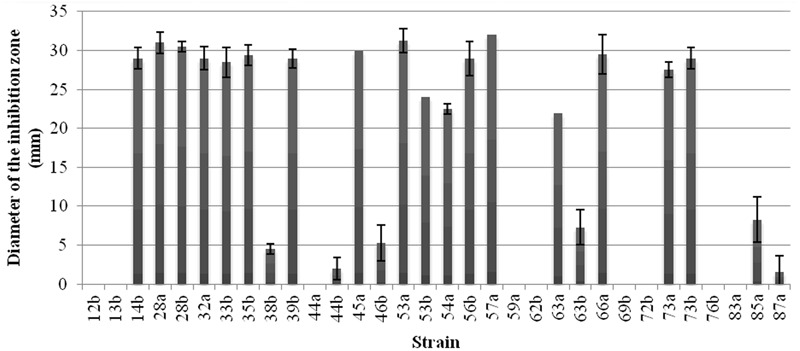
Production of the antibacterial compounds by the *Bacillus* strains. Inhibition zone, in mm, against *A. tumefaciens* C58 were recorded. Vertical bars represent standard errors of the means.

The bacterial strains were subjected to the production of antibacterial compounds in OM medium and then tested against *A. tumefaciens* C58 as indicator strain by the well diffusion assay. The obtained results showed that 17 strains produced antibacterial compounds leading to inhibition diameter above 20 mm (**Figure 2**). Nine strains out of the thirty-two do not produce antibacterial compounds against *A. tumefaciens* C58 in these conditions.

The antibacterial activity against *A. tumefaciens* correlated with the presence of the lipopeptide genes (*p* < 0.05). It was also significantly related with the presence of *bmyB* gene (*p* = 0.002). There was no association between this activity and the presence of the *sfp* gene (*p* = 0.429) and at the limit of association with the *ituC* gene (*p* = 0.046). Moreover, a correlation between the antibacterial activity and the hemolytic one was observed (*p* = 0.005).

#### Effect of Proteinase K on Antibacterial Activity

In order to determine the nature of antibacterial compounds produced against *A. tumefaciens* by the different strains, their sensitivity to the proteinase K was tested. Comparison of growth inhibition of the indicator strain in the presence of the supernatants treated or not with the proteinase K showed no significant differences for all the tested strains. This suggests that the bioactive compounds of the studied *Bacillus* strains are not of proteinic nature.

### *In Vivo* Biocontrol Test of the Antagonistic Strains toward *A. tumefaciens*

**FIGURE 3:**
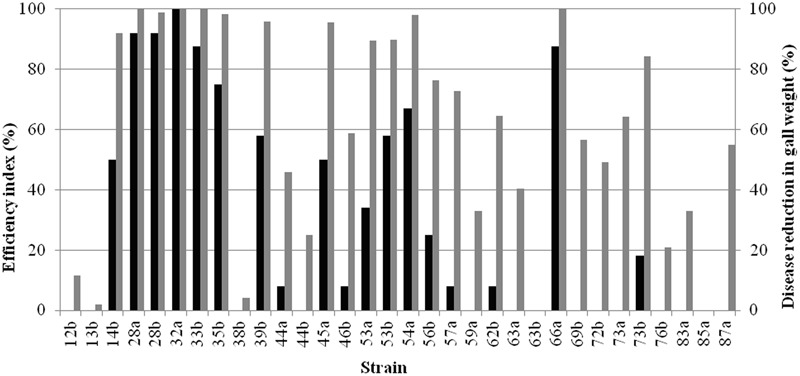
Efficiency of bacterial strains in the control of crown gall disease induced by *A. tumefaciens* C58. Biocontrol index (black bars) and disease reduction in gall weight (grey bars) were recorded.

Tests on tomato plants were conducted to assess the ability of the strains to reduce symptoms caused by *A. tumefaciens* strain C58 *in vivo*. Data were expressed as efficiency index and disease reduction in gall weight (**Figure 3**). Potent strains exhibiting the highest efficiency indices and disease reduction in gall weights were 28a, 28b, 32a, 33b and 66a.

**FIGURE 4:**
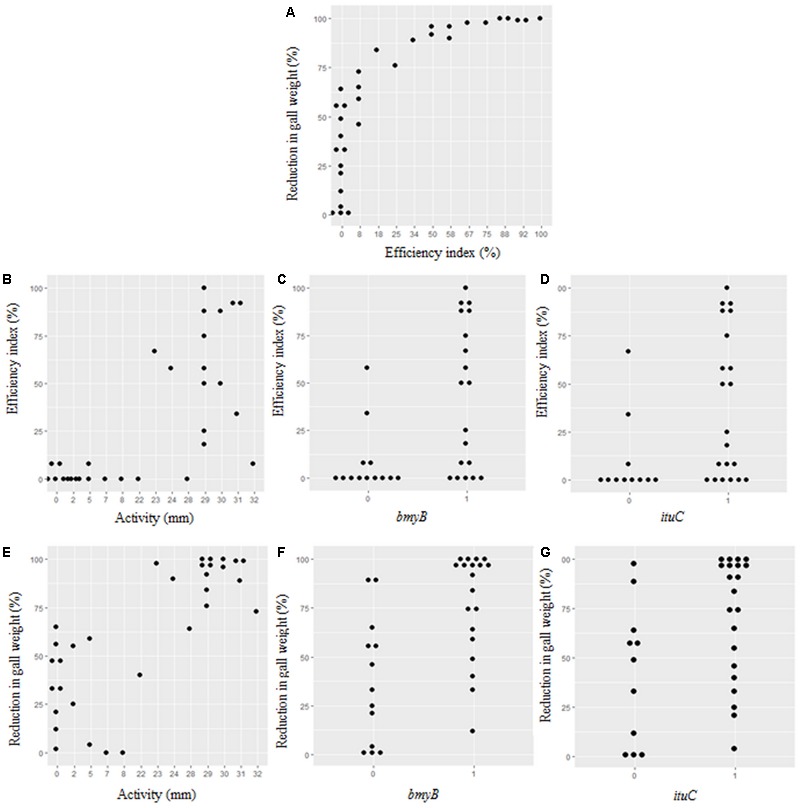
Correlations between the antibacterial activity, the presence of the *bmyB* and the *ituC* genes, the biocontrol index and the disease reduction in gall weight. **(A)** Correlation between reduction in gall weight and efficiency index. **(B–D)** Correlations between efficiency index and activity, *bmyB* and *ituC* genes, respectively. **(E–G)** Correlations between reduction in gall weight and activity, *bmyB* and *ituC* genes, respectively.

A high correlation between the efficiency of the biocontrol and the reduction of in gall weight (*p* = 0.000) was observed (**Figure 4A**). A high correlation between the efficiency of the biocontrol (*p* = 0.000) and the reduction of in gall weight (*p* = 0.000) with the antibacterial activity *in vitro* was also noticed (**Figures 4B,E**). Strains with high antibacterial activity showed high percentage of disease reduction of more than 60% (**Figure 4E**), although that for some of them low efficiency indices were observed (**Figure 4B**). On the other hand, strains with low activity allowed some reduction in gall weight reaching in the best cases 60% but their efficiency indices were low (**Figure 4E**).

Moreover, there was a strong correlation between the efficiency index (*p* = 0.004), the reduction in gall weight (*p* = 0.000) and the antibacterial activity (*p* = 0.002) with the presence of the *bmyB* gene. This was also the case for the presence of the *ituC* gene, except for the antibacterial activity *in vitro* (*p* = 0.013, *p* = 0.021 and *p* = 0.046, respectively). The distributions of the efficiency index and the reduction in gall weight in relation to the results of these two genes are represented in **Figures 4C,D,F,G**.

The antibacterial activity *in vivo* was also correlated to the presence of all the lipopeptide genes (*p* = 0.000). Furthermore, a correlation between the efficiency index and the hemolysis was observed (*p* = 0.021).

### Relationships between the Studied Variables

#### Principal Component Analysis

**FIGURE 5:**
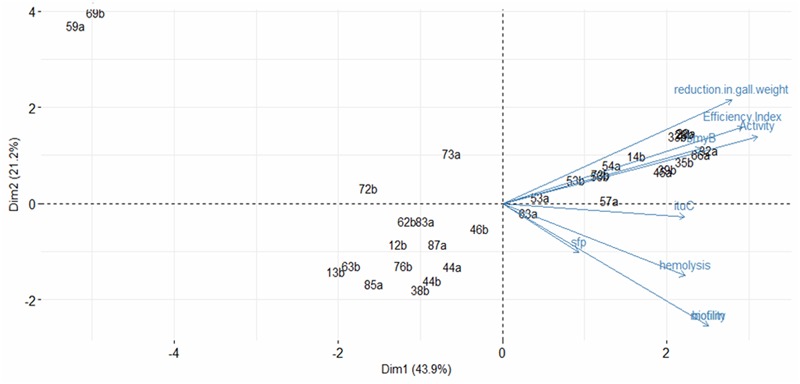
Principal component analysis profiles based on the antibacterial activity *in vitro*, the biocontrol efficiency, the reduction in gall weight, the presence of the *bmyB*, the *ituC* and the *sfp* genes, the hemolysis, the motility and the biofilm formation. The figure includes also the presentation of the strains. Percentage of variation accounted by each axis is indicated in parentheses.

The principal component analysis performed, using the studied variables, suggests two main groups of variable defined by two dimensions (**Figure 5**). The first dimension was represented by the activity *in vitro* (0.831), the efficiency Index (0.782), the reduction in gall weight (0.746) and the presence of the *bmyB* gene (0.647) whereas the second dimension was mainly represented by biofilm formation (-0.681) and motility (-0.681). The first dimension explains the major part of variation representing 43.9% of total variation, while the second accounts for 21.2% of variation. Globally, the two dimensions explain 65.1% of the variability. Plotting individuals indicated that strains with biocontrol ability are located in the right whereas those without biocontrol effect are located in the left.

#### Two-Way Hierarchical Cluster Analysis

**FIGURE 6:**
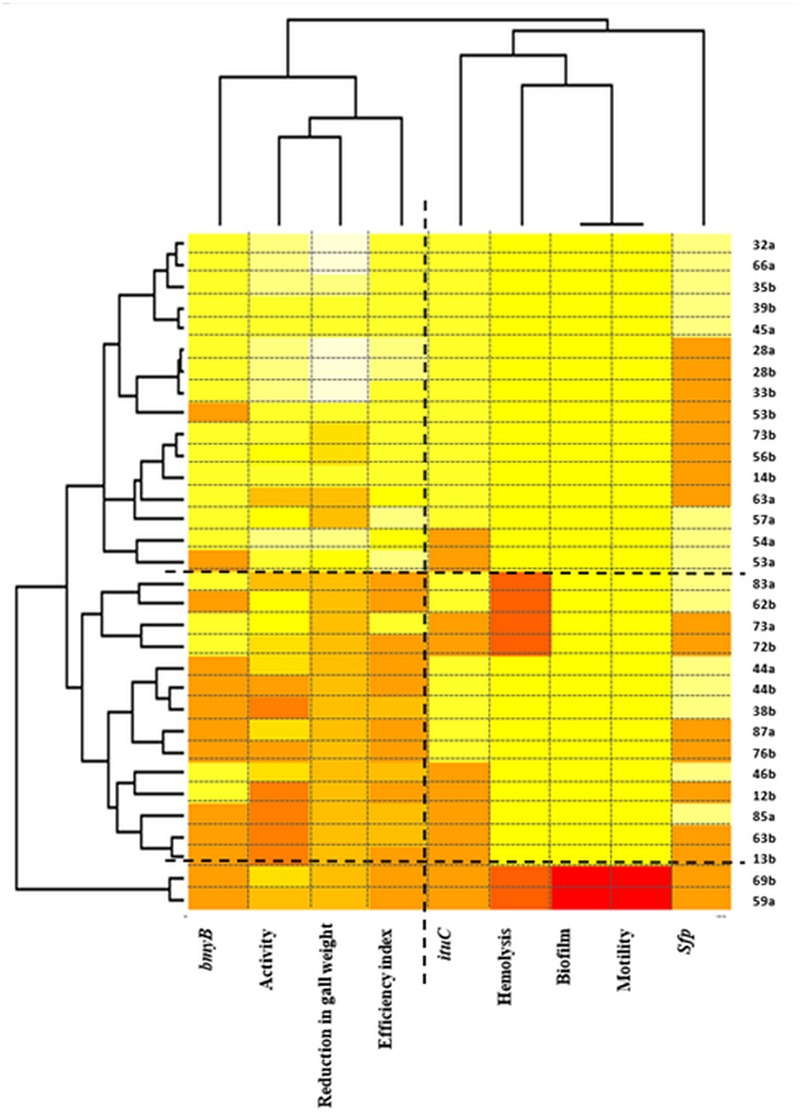
Two-way hierarchical clustering of *Bacillus* isolates and the various traits (the antibacterial activity *in vitro*, the biocontrol efficiency, the reduction in gall weight, the presence of the *bmyB*, the *ituC* and the *sfp* genes, the hemolysis, the motility and the biofilm formation). The colours show the proportion of the bands present at every designated band location. Colour indicates relative concentration: white, increased; red, decreased; from red to white: gradually increasing concentration.

Two-way hierarchical cluster analysis was performed to assess the relationship between the strains and the studied traits to identify which of these latter are the most important (**Figure 6**). The first dimension allowed classifying strains according to the various studied traits. Three clusters were defined. The first presented strains with high biological control ability indicated by high biocontrol efficiency indices and high reduction in gall weights but also with high activity *in vitro* and with the presence of the *bmyB* gene. The second and the third cluster represent strains with lower performances regarding the biocontrol ability. The third cluster includes only two strains 69a and 59a that were also characterised by the absence of all the biosynthetic lipopeptide genes, the antibacterial activity, the hemolysis, the biofilm formation and the motility.

Regarding the second dimension, two main clusters were defined confirming the results obtained by the correspondence analysis. The first grouped the biocontrol efficiency index, the reduction in gall weight, the antibacterial activity *in vitro* and the presence of the *bmyB* gene whereas the second grouped all the remaining traits.

The classification using hierarchical two-way cluster analysis correlated well with the results observed by the principal component analysis for both traits and strains. This confirms the accuracy of the significant traits to correctly select strains with biocontrol ability.

### Characterization of the Potent Strains

#### Isolation and Identification of Lipopeptides Using HPLC Analysis

Lipopeptides produced by the five potent *Bacillus* strains were analysed by HPLC analysis. According to the obtained results, strains 28b, 32a and 66a produced the surfactin and the iturin families contrarily to strains 28a and 33b that produced only surfactin in the OM medium (**Table 3**). Surprisingly, although the surfactin was produced by all strains, only strains 32a and 66a allowed the amplification of the *sfp* gene. Similarly, while the *ituC* and the *bmyB* genes were detected in all the five strains, the production of the iturinic family was only detected in strains 28b, 32a and 66a.

**Table 3:** Detection of lipopeptides in the potent strains.

	Surfactin	Iturin
28a	+	-
28b	+	+
32a	+	+
33b	+	-
66a	+	+

#### Identification of Selected Antagonistic Bacteria

Amplification and sequencing of the 16S rRNA was performed only for strains 28a, 28b, 33b and 66a, as strain 32a was previously identified as *B. amyloliquefaciens* (Ben Abdallah et al., 2015). Sequences were deposited in GenBank under accession numbers KY828470, KY828471, KY828472 and KY828473, respectively. Sequence analysis of the partial 16S rDNA gene indicated that all the strains belong to the *Bacillus* genus. Strains 28b and 33b were identified as *B. subtilis* whereas strains 28a as *B. amyloliquefaciens*. However, the discrimination of strain 66a remained ambiguous as sequence homology showed that it can be either *B. amyloliquefaciens* or *B. subtilis*.

## Discussion

This article describes the study of a collection of 32 *Bacillus* strains both *in vitro* and *in vivo* toward pathogenic *A. tumefaciens* strain C58 in order to improve the selection procedure of effective biological control agents against crown gall disease. All strains were investigated for the various traits so that correlations between them could be assessed. It is important to note that carrying out the *in vivo* test for all the 32 studied strains represents the main strength of our work due to the difficulty of its realisation and to the high number of analysed strains. The principal component analysis and the two-way hierarchical cluster analysis correlating the studied traits with the biocontrol ability of *Bacillus* strains were used. Three groups of *Bacillus* strains were detected. The first included strains with high antibacterial activity *in vitro* associated with the presence of the *bmyB* gene that showed biocontrol ability against the crown gall disease. The second and the third group, on the contrary, present strains with no or low activity *in vitro* with the absence of the *bmyB* gene and without or with low biocontrol ability. The most relevant features for the differentiation of these groups related to their biocontrol ability were the antibacterial activity, followed by the *bmyB* gene. Our results showed that antibiosis has a high correlation with the biocontrol efficiency and with the reduction in gall weight. Many strains, with high antibacterial activity, were effective in increasing efficiency index and in reducing significantly the weight of galls. Moreover, the majority of strains (23 out of the 32) allowed disease reduction in gall weight, reflecting significantly reduced disease severity in the affected plants. The biocontrol assays also demonstrated a clear and significant segregation between producers and non-producers of the antibacterial compounds. Contrarily to low producers, strong producer strains *in vitro* probably produce the required amount of lipopeptides *in vivo* for the expression of their biocontrol activity. An efficient production of lipopeptides is therefore important for the biocontrol activity of *Bacillus* strains as well as for their global fitness in natural habitat including the colonisation and the biofilm formation abilities. These are important mechanisms involved in biological control of several plant diseases (Bais et al., 2004; Ongena and Jacques, 2008). The grouping based on the principal component analysis and the two-way hierarchical cluster analysis could be an efficient tool to minimise the number of strains to be studied during the selection of a biological control agent allowing the discovery of new and effective strains. In fact, antibiosis could be taken as a primary step for the selection of biocontrol agents against *A. tumefaciens*. This step could allow discarding almost half of the investigated strains due to the absence of *in vitro* activity against *A. tumefaciens*. Further selection has to be performed on tomato plants *in vivo*. This strategy could be more efficient as it would reduce the cost, time, and efficacy. This second selection step is also primordial as it would allow discarding strains with moderate performance regarding the biocontrol efficiency but with high antibacterial activity such as strains 73b, 56b, 63a and 57a.

Study of the lipopeptide biosynthetic genes indicated that *ituC* and *bmyB* genes were more frequently detected than *sfp*. Moreover, there was a tendency for strains to harbour more than one gene. This was expected as those without activity (those without antibiotic genes or with only one gene), were discarded in the first screening when constituting our strain collection. The widespread presence of several operons involved in antimicrobial peptide biosynthesis in the genomes of *Bacillus* biocontrol agents of plant diseases, such as *B. amyloliquefaciens* FZB42, was reported to account for up to 12% of the genomes (Koumoutsi et al., 2004; Chen et al., 2007; Arguelles-Arias et al., 2009). The antimicrobial activity *in vitro* was related either to the presence of lipopeptide biosynthetic genes simultaneously or to the coproduction of multiple lipopeptides (Joshi and McSpadden Gardener, 2006; Ramarathnam et al., 2007; Mora et al., 2012, 2015). In fact, when different families of lipopeptides are co-produced, their interaction can become synergistic and enhances each of their respective activities (Romero et al., 2007; Kim et al., 2010; Deravel et al., 2014). Regarding the control of crown gall disease, strains producing the three lipopeptide families were found to be effective (Ben Abdallah et al., 2015; Frikha-Gargouri et al., 2017). Moreover, the protection *in vivo* was linked to the production of lipopeptides that was suggested as a factor for disease suppression (Ben Abdallah et al., 2015; Frikha-Gargouri et al., 2017). These were the only compounds detected in the rhizosphere and in different parts of plants among the other antimicrobial compounds produced by *Bacillus* biocontrol strains (Toure et al., 2004; Thomashow et al., 2008; Kinsella et al., 2009). The investigation of the presence of lipopeptide biosynthetic genes indicated that the antibacterial activity against *A. tumefaciens* was significantly related to the presence of *bmyB* and the *ituC* genes. Mora et al. (2015) reported that the *bmyB* gene was significantly related to the activity against *Rhizobium radiobacter*. These authors and others suggested that non-ribosomal peptide synthetase genes may be used as markers for the identification and selection of novel biocontrol agents from environmental samples (Giacomodonato et al., 2001; Mora et al., 2015). Besides the antibacterial activity *in vitro*, the detection of the *bmyB* gene could also be used for the selection of *Bacillus* biocontrol agents against *A. tumefaciens* due to its high association with the biocontrol ability. On the contrary, the presence of the *ituC* gene seems to be less suitable for such selection. Moreover, accessing the presence of the *ituC* and the *sfp* genes, the hemolysis, the biofilm formation and the motility in the screening of biocontrol agents are of limited values.

The relationship between genes and products of the five potent strains was investigated showing generally low correlation. In fact, although the surfactin was produced by all strains, only strain 32a and 66a allowed the amplification of the *sfp* gene indicating low sensitivity of PCR probably due to mutations. Additionally, although the *ituC* and the *bmyB* genes were detected in the five strains, the production of the iturinic family was only detected in strains 28b, 32a and 66a. This could be related to the differential production of lipopeptides according to the growth medium used (Mukherjee and Das, 2005; Fahim et al., 2012); but also to mutations. Similar conclusion was advanced by Mora et al. (2015) for the detection of the *bmyB* and the *srfAA* and their corresponding products. The five successful strains showed differential patterns of lipopeptide production that are surfactin only for strains 28a and 33b and surfactin and iturin for strains 28b, 32a and 66a. Interestingly, surfactin was the only lipopeptide produced by all these potent strains. Its presence is in agreement with its only detection in the methanolic extract of the active fraction in the thin layer chromatography extract of strain 39b (Frikha-Gargouri et al., 2017).

Some results related to the principal component analysis and the two-way hierarchical cluster analysis have to be highlighted. First, two strains were located outside of the groups of *Bacillus* strains (69a and 59a) in the principal component analysis (at the upper left side) and the two-way hierarchical cluster analysis (the third cluster composed of these two strains only). They were found to belong to the *Paenibacillus* genus (Data not shown). Only these strains were unable to form biofilm and to colonise the medium. Strain 69a showed a reduction in gall weight although the absence of any biosynthethic genes nor any other trait. Its biocontrol ability could be related to another mechanism of action than antibiosis. Second, regarding antibiosis, only strain 63a had low activity *in vitro* but had high efficiency index. As for strain 69a, other mechanisms of action could be involved. Only strain 73a had a relatively high antibacterial activity *in vitro* but showed limited biocontrol ability especially a low efficiency index. Effective compounds in this strain could be secreted efficiently *in vitro* in the OM medium but not *in vivo*, contrarily to those produced by all the other strains. Third, regarding the *bmyB* gene, its detection showed a higher discordance with the biocontrol ability when compared to the detection of the antibacterial activity *in vitro*. In fact, two strains were classified in the biocontrol group did not allow the amplification of the *bmyB* gene whereas five strains that allowed such amplification were present in the lower performance groups regarding the biocontrol ability. Among these five strains, three of them allowed the reduction in gall weight. This corroborated the superiority of the antibacterial activity over the detection of *bmyB* gene when selecting effective biocontrol agents against crown gall disease.

## Conclusion

The two way hierarchical cluster and the correspondence analysis tools were used to study the relationships between *Bacillus* strains and the various traits and to identify the most important ones to be considered in the screening of biocontrol agents against *A. tumefaciens* strains. Our results suggested that antibiosis and the presence of the *bmyB* gene could be used for the preliminary screening of biocontrol strains against crown gall disease. Further selection has to be performed on tomato plants *in vivo*. Five potent *Bacillus* strains showed important biological control ability against crown gall disease making them promising candidates to be included in biocontrol programmes. Although antibiosis seems to be the mechanism involved in their biocontrol activity, further analysis of their modes of action is currently being undertaken.

## Author Contributions

OFG designated the study, performed experimental work and statistical analysis, and wrote the manuscript. DBA contributed in the HPLC analysis. IB contributed to the *in vitro* and the *in vivo* experiments. ST critically read and approved the final version of the manuscript and participated in the study design.

## Conflict of Interest Statement

The authors declare that the research was conducted in the absence of any commercial or financial relationships that could be construed as a potential conflict of interest.
